# αE-catenin-dependent mechanotransduction is essential for proper convergent extension in zebrafish

**DOI:** 10.1242/bio.021378

**Published:** 2016-09-09

**Authors:** Mitchell K. L. Han, Esteban Hoijman, Emily Nöel, Laurence Garric, Jeroen Bakkers, Johan de Rooij

**Affiliations:** 1 Molecular Cancer Research, Center for Molecular Medicine, University Medical Center Utrecht, Universiteitsweg 100, Utrecht 3584CG, The Netherlands; 2 The Hubrecht Institute for Developmental Biology and Stem Cell Research and University Medical Center Utrecht, Uppsalalaan 8, Utrecht 3584CT, The Netherlands; 3 Department of Medical Physiology, University Medical Center Utrecht, Yalelaan 50, Utrecht 3584 CM, The Netherlands

**Keywords:** Zebrafish, Cadherin mechanotransduction, Alpha-catenin, Convergent extension, Embryogenesis

## Abstract

Cadherin complexes mediate cell-cell adhesion and are crucial for embryonic development. Besides their structural function, cadherin complexes also transduce tension across the junction-actomyosin axis into proportional biochemical responses. Central to this mechanotransduction is the stretching of the cadherin-F-actin-linker α-catenin, which opens its central domain for binding to effectors such as vinculin. Mechanical unfolding of α-catenin leads to force-dependent reinforcement of cadherin-based junctions as studied in cell culture. The importance of cadherin mechanotransduction for embryonic development has not been studied yet. Here we used TALEN-mediated gene disruption to perturb endogenous αE-catenin in zebrafish development. Zygotic α-catenin mutants fail to maintain their epithelial barrier, resulting in tissue rupturing. We then specifically disrupted mechanotransduction, while maintaining cadherin adhesion, by expressing an αE-catenin construct in which the mechanosensitive domain was perturbed. Expression of either wild-type or mechano-defective α-catenin fully rescues barrier function in α-catenin mutants; however, expression of mechano-defective α-catenin also induces convergence and extension defects. Specifically, the polarization of cadherin-dependent, lamellipodia-driven cell migration of the lateral mesoderm was lost. These results indicate that cadherin mechanotransduction is crucial for proper zebrafish morphogenesis, and uncover one of the essential processes affected by its perturbation.

## Abstract (precis)

**Summary:** Cadherin adhesions transduce tension across the junction-actomyosin axis into proportional biochemical responses via α-catenin. Here we show for the first time how this function of the cadherin complex is important during zebrafish morphogenesis.

## INTRODUCTION

During embryonic development, tissue shape and organization is established by a series of complex cell migrations and rearrangements. These developmental cell-movement processes are largely dependent on dynamic intercellular adhesions (Martin and Goldstein, 2014). Central to cell-cell adhesion are the classical cadherins, such as E-Cadherin, which connect cells through homotypic interactions between their extracellular domains (Leckband and de Rooij, 2014). Through their cytoplasmic tails, classical cadherins bind p120- and β-catenin, the latter of which in turn associates with the actin-binder α-catenin. Together they form the cadherin complex which assembles in structures known as adherens junctions (AJs), which are essential for the maintenance of barriers in epithelial tissues (Niessen, 2007). Besides in barrier forming AJs, cadherins also cluster in a different type of cell-cell junction, called a focal adherens junction (FAJ), that is more dynamic and particularly apparent during tissue remodeling processes (Huveneers and de Rooij, 2013). By coupling the actomyosin cytoskeletons of neighboring cells through AJs or FAJs, cadherins mediate the formation of a contractile network of cells. Thus cadherins are central to many aspects of cell-cell adhesion and tissue dynamics.

How strongly cadherin-based junctions are involved in tissue shape generation is especially apparent during gastrulation, the developmental process in which germ layer progenitor cells migrate and rearrange their cell-cell contacts to achieve tissue morphogenesis. One such movement in zebrafish is epiboly, the event in which several layers of cells spread and migrate over the yolk cell (Bruce, 2016). Here the loosely packed deep cells intercalate radially outward into the outer-most epithelial sheet known as the EVL, thereby pushing their neighbors and contributing to thinning of the deep cell layers during epiboly progression. Both embryos in which E-cadherin- and α-catenin was knocked down with anti-sense morpholinos, as well as E-cadherin mutant embryos, show epiboly delay (Kane et al., 2005; Schepis et al., 2012; Shimizu et al., 2005). This is possibly due to reduced adhesion of the deep cells to the EVL, which is mediated by cadherin adhesion. Moreover, in these embryos it was observed that deep cells failed to integrate into more superficial layers, and also dropped back into lower layers. Cadherins have also been implicated in the regulation of cell intercalation in *Xenopus* (Chen and Gumbiner, 2006; Marsden and DeSimone, 2003) and *Drosophila* (Rauzi et al., 2010).

Another concurrent morphogenetic process in zebrafish embryos in which the importance of cell-cell junctions is apparent is convergent extension (CE), which facilitates body axis elongation (Tada and Heisenberg, 2012; Yin et al., 2009). During CE, the germ layer progenitor cells undergo a combination of migration and cell intercalation events to converge towards the dorsal midline, while simultaneously elongating along the anterior-posterior axis (Yin et al., 2008). At the shield stage, mesendoderm progenitors, which later give rise to the prechordal plate, internalize at the blastoderm margin as single cells, but subsequently form a highly cohesive cluster of cells that migrates along the overlying epiblast towards the animal pole, thereby contributing to tissue extension along the anterior-posterior axis. Both the cohesiveness of the cluster, the migration along the overlying epiblast (Montero et al., 2005; Ulrich et al., 2005), as well as its directionality (Dumortier et al., 2012) were shown to be dependent on E-cadherin adhesion. Convergence is mainly driven by the migration of mesoderm cells to the dorsal midline (Sepich et al., 2005), and is dependent on both E- and N-cadherin (Arboleda-Estudillo et al., 2010; Warga and Kane, 2007). Furthermore, a BMP-induced gradient of N-cadherin-dependent cell-cell adhesion was found to regulate the lammellipodial dynamics underlying the dorsal migration of lateral mesoderm (von der Hardt et al., 2007).

Recent studies show that the cadherin complex not only serves as a structural anchor between cell adhesions and the actomyosin cytoskeleton, but is also an active site of mechanotransduction as increasing tensile forces on cadherin-based junctions induce biochemical changes, which among others lead to force-dependent reinforcement of the adhesion (Bazellières et al., 2015; Kannan and Tang, 2015; le Duc et al., 2010). A key element in cadherin mechanotransduction is α-catenin, which undergoes a conformational change when under tension. This change alters the conformation and orientation of central domains in α-catenin (Pokutta et al. 2002). The best known consequence of this is the recruitment of the protein vinculin to tensile cell-cell junctions (Yonemura et al., 2010). Force-dependent recruitment of vinculin is particularly apparent during tissue remodeling processes, where dynamic FAJs are formed (Huveneers et al., 2012). Replacement of the mechanosensitive vinculin-binding domain in α-catenin by a homologous domain from vinculin (α-catenin-ΔVBS) perturbed vinculin recruitment, in addition to mechanosensing and junction reinforcement during epithelial barrier formation (Twiss et al., 2012). Human umbilical vein endothelial cells (HUVEC) expressing α-catenin-ΔVBS show more severe junction breakage during junction remodeling (Huveneers et al., 2012). It should be noted that besides vinculin, additional proteins (α-actinin, formin, afadin) were mapped to interact to the perturbed domain in this mechanosensing-defective α-catenin (Kobielak et al., 2004; Nieset et al., 1997; Pokutta et al., 2002) and could therefore be of equal importance to cadherin mechanotransduction.

While the importance of the structural role of the cadherin complex has been well established, the importance of cadherin mechanotransduction during the development of a live organism has not been investigated. To address this we used zebrafish morphogenesis as a model system. We first generated αE-catenin-deficient zebrafish using TALEN gene engineering. Homozygous mutant embryos showed defects in epithelial barrier formation and subsequent lethality during somitogenesis. These defects could be fully rescued by restoring α-catenin function through mRNA injection. We then specifically disrupted cadherin mechanotransduction, while maintaining its structural function, by injecting the α-catenin-ΔVBS construct. The resulting embryos showed no barrier defects and survived somitogenesis, but now exhibited defects in convergent extension – underlying cell migrations that caused severely perturbed morphogenesis. Zooming in on one of these migration processes by live cell imaging, we observed that the dorsal migration of the lateral mesoderm cells was reduced. In particular, we found defects in the polarization of cadherin-dependent lammellipodial adhesion in these cells. Thus, α-catenin mediated cadherin mechanotransduction is essential for cadherin-dependent cell migration during zebrafish morphogenesis.

## RESULTS

### Generation of *ctnna1*-deficient mutants

**Fig. 1.**
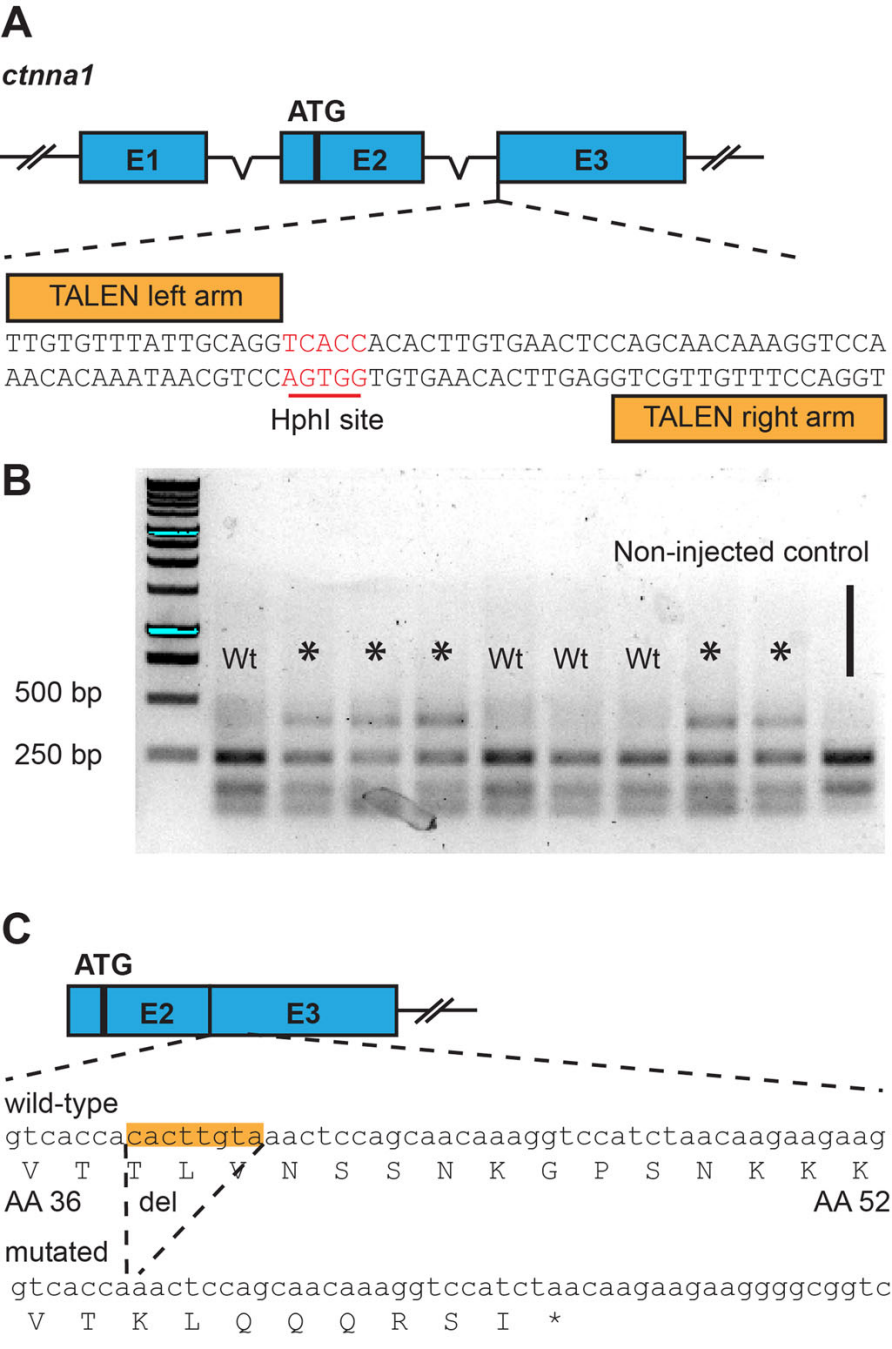
**Generation of *ctnna1* mutants using TAL-effector nucleases (TALENs).** (A) Schematic representation of the endogenous *ctnna1* locus which was targeted by TALENs at the intron2-exon 3 boundary. The TALEN arms flank a restriction enzyme recognition site (highlighted in red) used for screening mutant alleles through restriction fragment length polymorphism (RFLP) analysis. (B) RFLP analysis of embryos injected with *ctnna1*-specfic TALENs. Uncleaved bands showing successful TALEN activity are marked by an asterisk. Wt, wild type. (C) The sequence of the *cttna1* mutant allele and the consequence on protein translation.

To investigate the role of αE-catenin-dependent cadherin mechanotransduction during zebrafish morphogenesis, we first generated an αE-catenin (*ctnna1*) deficient mutant. We disrupted the endogenous *ctnna1* locus using TAL-effector nucleases (TALENs) (Cermak et al., 2011) by targeting the Intron 2-Exon 3 boundary (Fig. 1A). The TALEN cleavage activity was assessed using restriction fragment length polymorphism (RFLP) analysis, which is facilitated by the presence of an HphI restriction enzyme recognition sequence in the cleavage site. The target site was amplified from genomic DNA of *ctnna1* TALEN-injected embryos, and the resulting PCR product was subjected to RFLP analysis (Fig. 1B). Samples from non-injected controls show bands of approximately 140 bp and 240 bp after HphI digestion, while samples from TALEN-injected embryos may show a third, uncleaved band at around 400 bp. These uncleaved bands point to the induction of indel mutations which disrupt the restriction enzyme recognition site. Sixty four out of 94 embryos injected with the *ctnna1* TALEN showed targeted gene disruption at the endogenous locus. While no bi-allelic targeting was observed, even with higher dosages of TALEN mRNA (data not shown), we can thus conclude that the TALEN is able to efficiently cleave the endogenous locus at the intended target site.

Next, TALEN-injected embryos were raised to adulthood and then genotyped by fin clipping to identify potential founders. Of 54 fish screened we could identify 25 fish with somatic *ctnna1* mutations. Individual mutant fish were outcrossed to wild-type fish and the offspring checked for germline transmission of mutations. While individuals showed different germline transmission rates, of the 87 embryos screened from crosses of potential founders with wild-type, 16 embryos (18%) were found to be heterozygous for TALEN-generated *ctnna1* mutant alleles. TALENs induce double-stranded breaks at the target site, which due to the error-prone non-homologous end-joining mechanism results in indel mutations (Cermak et al., 2011). Only deletion Δ8 caused a non-sense mutation, leading to a premature stop codon after Threonine37, which resides 5′ to the β-catenin-binding domain and actin-binding domain (Fig. 1C). The α-catenin fragment resulting from this mutated allele contains no known interaction sites and is thus most likely a null mutation. Therefore from this point on, the line carrying the *ctnna1^Δ8^* allele is designated as *ctnna1*^hu10414^ and is the main *ctnna1* mutant described further. In conclusion, we generated an αE-catenin null allele which we used to investigate the effect of loss of αE-catenin by incrossing heterozygous mutants.

### Depletion of zygotic *ctnna1* causes loss of epithelial barrier function

**Fig. 2.**
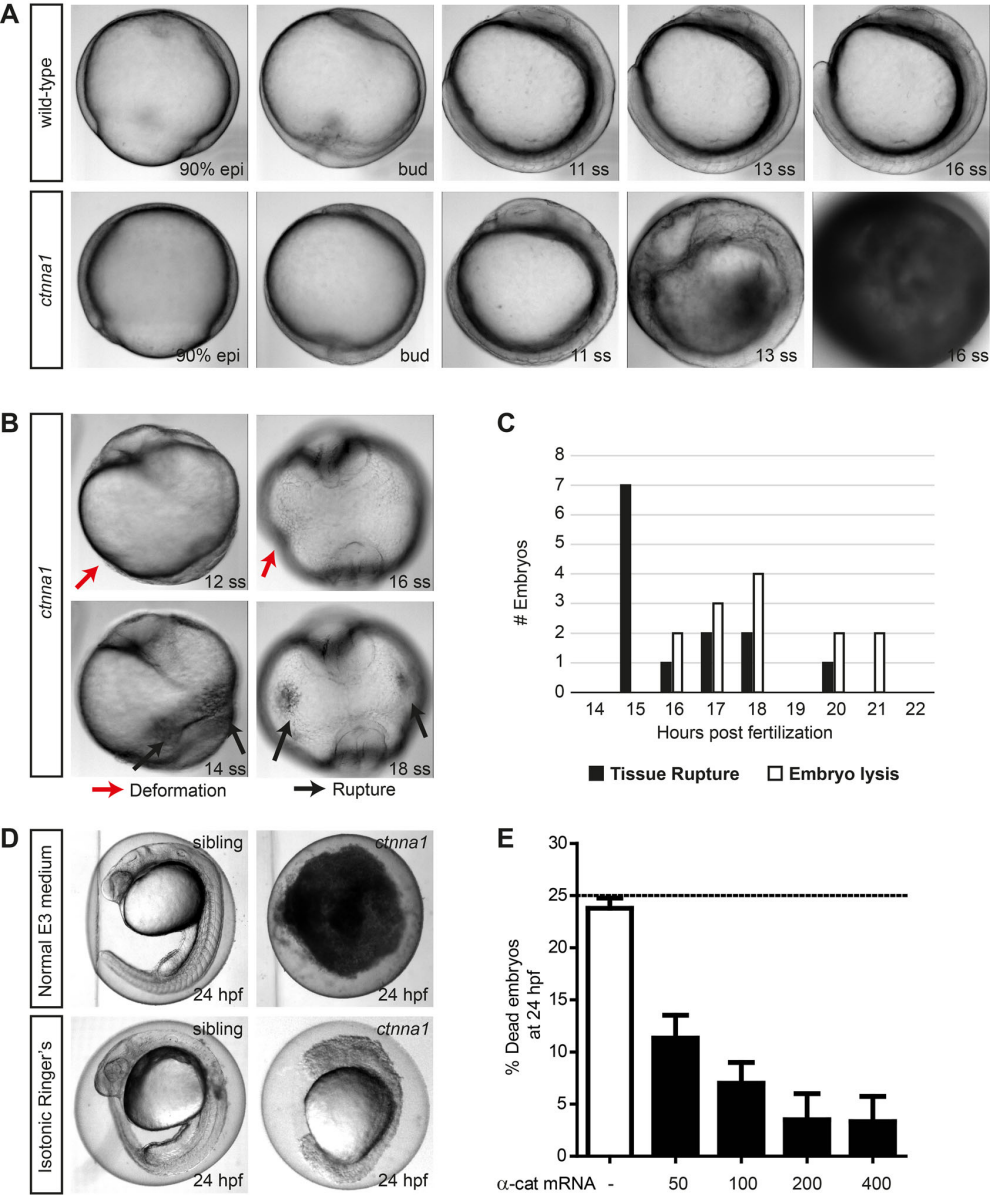
**Zygotic *ctnna1* mutants show defects in epithelial barrier functioning.** (A,B) DIC images from a time-lapse series of zygotic *ctnna1* mutants and wild-type siblings imaged from 90% epiboly until 21 somite stage. Lateral view in (A) shows embryo lysis in zygotic *ctnna1* mutants during somitogenesis. Anterior views of zygotic *ctnna1* mutants in (B) show deformation (red arrows) and tissue rupture (black arrows) occurring before embryo lysis. (C) Quantification of the time of rupture and subsequent embryo lysis in zygotic *ctnna1* mutants (*n*=13 embryos from three independent experiments). (D) Phenotype at 24 hpf of zygotic *ctnna1* mutants and siblings grown in regular E3 medium, and embryos transferred to isotonic Ringer's buffer at 50% epiboly. (E) Rescue of embryo lysis in *ctnna1* mutants using increasing concentrations of α-catenin-GFP mRNA. The dashed line indicates the expected mortality of non-injected incrossed heterozygous *ctnna1* mutants based on Mendelian genetics. Data represent three independent experiments, *n*>120 embryos per condition. Data are represented as the mean±s.e.m.

Zygotic *ctnna1* mutants show no major defects during cleavage or epiboly stages. In contrast, during somitogenesis, tissue rupture and subsequent swelling of the yolk was observed, leading to a striking embryonic lethal phenotype in which the yolk and cells of the embryo proper have undergone lysis (Fig. 2A, stills from time-lapse sequence, and Movie 1). Prior to embryo lysis, slight embryo deformations can be visible as indentations compared to the normally spherical morphology of the embryo at this stage (Fig. 2B, top). In addition, ruptured weak points in the external epiblast can be observed as black spots. This tissue rupturing did not occur in a fixed location in the embryo, but occurred in diverse regions in different embryos, such as on the lateral epithelium overlaying the yolk or near the forming tailbud (Fig. 2B, bottom). Furthermore, not all embryos undergo lysis at the same time (Fig. 2C), indicating that tissue rupture seemed not restricted to a particular tissue or developmental process but rather occurs stochastically due to gradual loss of tissue integrity. We reasoned that tissue rupture is caused by a perturbed epithelial barrier due to loss of functional adherens junctions. This in turn would increase the osmotic pressure and induce the observed swelling of the yolk. To test this, we transferred embryos at 50% epiboly stage from regular E3 embryo medium to isotonic Ringer's buffer. Under these conditions most mutants did not undergo tissue rupturing, but survived until after 24 hpf. However, unlike wild-type or heterozygous siblings, the cells of these *ctnna1* mutant embryos show a very rounded morphology at this time point, indicating a lack of functional adherens junctions (Fig. 2D). By injecting αE-catenin mRNA into offspring of incrossed *ctnna1* heterozygous mutants at the one-cell stage, we were able to rescue the embryo lysis seen in zygotic *ctnna1* homozygous mutants in a dose-dependent manner (Fig. 2E), further confirming that this phenotype is indeed caused by loss of α-catenin. Rescued *ctnna1* embryos survive and look indistinguishable from non-injected control embryos until at least 24 hpf, after which the outer epithelium starts to undergo lysis. This is most likely attributed to a gradual loss of injected α-catenin mRNA and subsequent loss of functional protein. These results demonstrate that *ctnna1* is essential for the maintenance of a functional epithelium, which in turn is crucial for zebrafish embryonic development.

### The mechanosensitive domain of αE-catenin is not necessary for the formation of a functional epithelial barrier

**Fig. 3.**
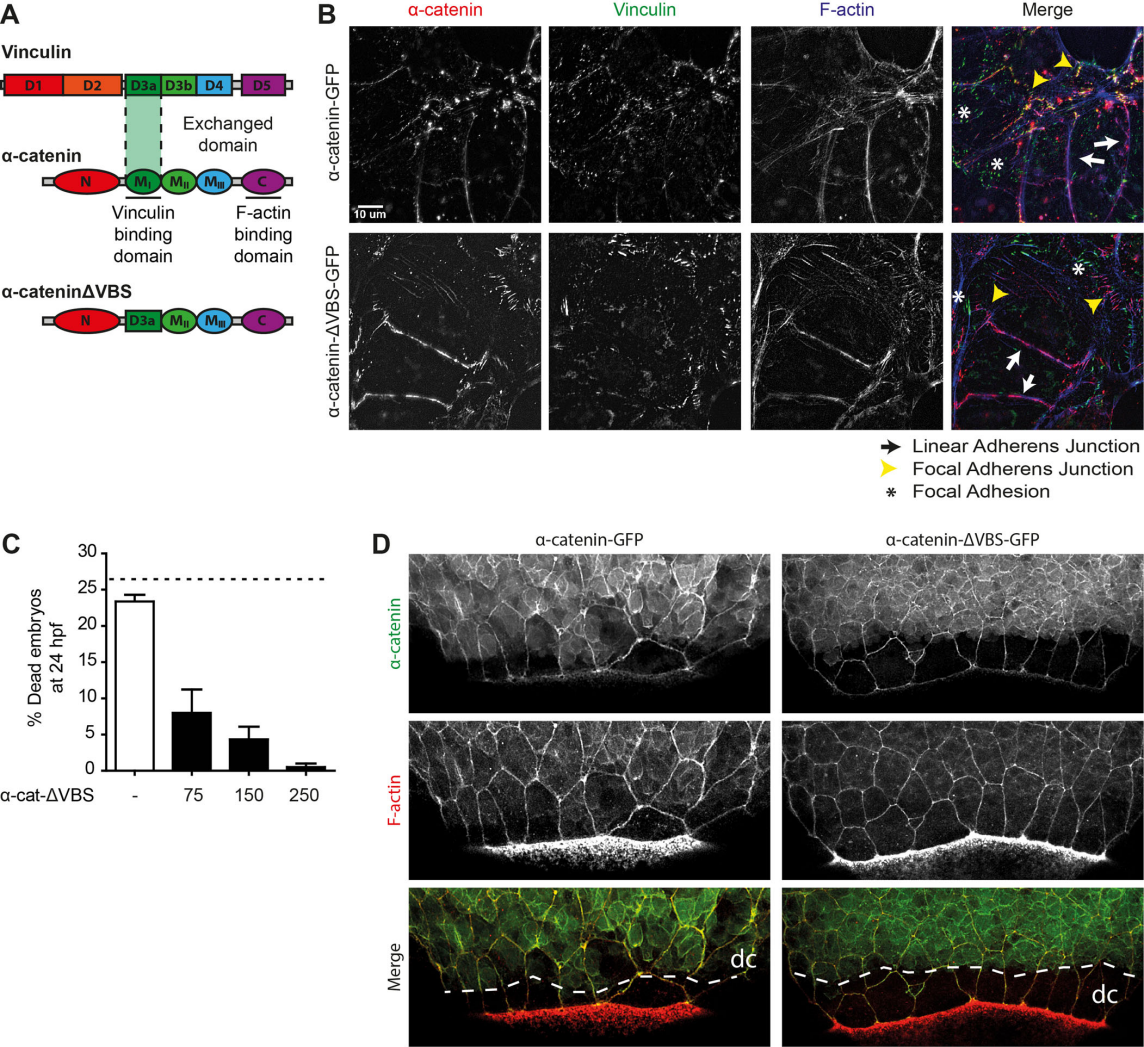
**The vinculin-binding domain in αE-catenin is not needed for barrier function.** (A) Schematic representation of the structures of α-catenin, vinculin and the exchanged domain in α-catenin-ΔVBS. The domain containing the vinculin binding site (VBS) was replaced by a homologous domain from vinculin itself, which does not bind vinculin. (B) Images of fixed α-catenin-deficient DLD1 R2/7 cells expressing zebrafish α-catenin-GFP (top) or α-catenin-ΔVBS-GFP (bottom) (both depicted in red), and stained for vinculin (green) and F-actin (blue). Linear adherens junction structures are highlighted by the white arrows, focal adherens junctions with the yellow arrowheads, and focal adhesions with white asterisks. (C) Rescue of embryo lysis in *ctnna1* mutants using increasing concentrations of α-catenin-ΔVBS-GFP mRNA. The dashed line indicates the expected mortality of non-injected incrossed heterozygous *ctnna1* mutants based on Mendelian genetics. Data represents three independent experiments, *n*>120 embryos per condition. Data is represented as the mean±s.e.m. (D) Whole-mount immunostaining of zebrafish embryos at 85% epiboly expressing either α-catenin-GFP (left) or α-catenin-ΔVBS-GFP (right) (both depicted in green) and stained for F-actin (red). The dashed white line marks the deep cell margin (dc) while the actin ring marks the EVL margin.

Tension-induced unfolding of α-catenin is a key element in cadherin mechanotransduction (Twiss et al., 2012; Yao et al., 2014). To interfere with cadherin mechanotransduction in adherens junctions, we generated an αE-catenin construct lacking mechanosensing capacity. Specifically, we exchanged the D3a domain containing the vinculin binding site (VBS) of zebrafish α-catenin with a structurally highly homologous domain. Coincidentally this domain is obtained from vinculin itself, as it is α-catenin's closest homologue [as has previously been described for mammalian α-catenin (Huveneers et al., 2012; Twiss et al., 2012)] (Fig. 3A). The mammalian version of this mutant protein, called α-catenin-ΔVBS, was shown to maintain barrier forming capacity in mammalian epithelial cell culture even though its mechanosensitive capacity (as measured by magnetic twisting cytometry) was completely lost (Twiss et al., 2012). Moreover, mammalian α-catenin-ΔVBS was shown to be incapable of binding vinculin even when fully activated (Huveneers et al., 2012). It was concluded from this previous work that the domain replacement resulted in a structurally intact protein that can function to link the cadherin complex to the actomyosin cytoskeleton. Conversely, α-catenin-ΔVBS fails to perform mechanotransduction because binding to key effectors is abolished due to the difference in primary sequence between the original D3a domain and the replacement.

To characterize zebrafish α-catenin and α-catenin-ΔVBS, we expressed GFP-tagged versions in α-catenin-deficient DLD1-R2/7 cells (that do not form any cell-cell junctions normally). As shown in Fig. 3B, both zebrafish α-catenin-ΔVBS-GFP and wild-type zebrafish α-catenin-GFP restore junction formation to a similar level (Fig. 3B, bottom). Furthermore, the mechanosensitive effector vinculin is recruited to wild-type α-catenin-induced junctions. More specifically, vinculin concentrates at those junctions that display a discontinuous morphology and are oriented perpendicular to the plane of cell-cell contact (demarcated by yellow arrowheads in Fig. 3B, top). We have previously called these junctions focal adherens junctions to convey their distinct appearance and association with radially oriented actin bundles, indicating that tension is applied to these junctions. Conversely, the α-catenin-induced junctions that are continuous within the plain of adhesion and likely experience less tension, which we call linear adherens junctions (demarcated by white arrows in Fig. 3B), recruited vinculin less efficiently. This closely mimics the vinculin recruitment to junctions induced by mammalian α-catenin in these cells (Huveneers et al., 2012; Twiss et al., 2012) and this shows that zebrafish α-catenin is functionally equivalent to mammalian α-catenin both in structural support of cadherin adhesion as well as in mechanosensing capacity. In contrast to wild-type α-catenin-GFP, α-catenin-ΔVBS-GFP does not induce the recruitment of vinculin to focal adherens junctions, as observed by the lack of overlap in the red (α-catenin) and green (vinculin) channels (Fig. 3B, bottom). This result confirms that also in the zebrafish isoform of α-catenin-ΔVBS-GFP mechanosensitive capacity is perturbed, while its structural function in cell-cell adhesion is preserved.

To investigate whether α-catenin-ΔVBS-GFP is able to support junction formation *in vivo*, we injected α-catenin-ΔVBS-GFP mRNA in the offspring of incrossed heterozygous *ctnna1* mutants. As shown in Fig. 3C, α-catenin-ΔVBS-GFP is able to rescue the embryonic lethal phenotype, caused by a defect in barrier maintenance, of zygotic *ctnna1* mutants in a dose-dependent manner. This shows that both α-catenin-ΔVBS-GFP can rescue the barrier defect similar to wild-type α-catenin and that the replacement of the VBS does not impede the maintenance of a functional epithelial barrier throughout early development. Indeed both α-catenin-GFP and α-catenin-ΔVBS-GFP properly and similarly localize to cell-cell junctions in the developing embryo. This can be observed in the enveloping layer (EVL) as well as in the underlying deep cells in the embryos shown in Fig. 3D and Fig. S2. Movie 2 shows additional evidence of junctional localization of α-catenin-GFP, and also demonstrates the difference in dynamics between EVL adherens junctions that are stable and linear and deep cell adherens junctions that are constantly remodeled and short-lived, similar to the FAJs that we have characterized in 2D cultured mammalian epithelia (see also Movie 5).

To conclude, we have generated an α-catenin-deficient zebrafish mutant as well as functional rescue constructs, which behave analogously to the mammalian α-catenin constructs previously described, and that allow us to specifically perturb mechanotransduction without interfering with the structural role of α-catenin in junctions.

### The mechanosensitive domain is essential for proper convergence extension movements

**Fig. 4.**
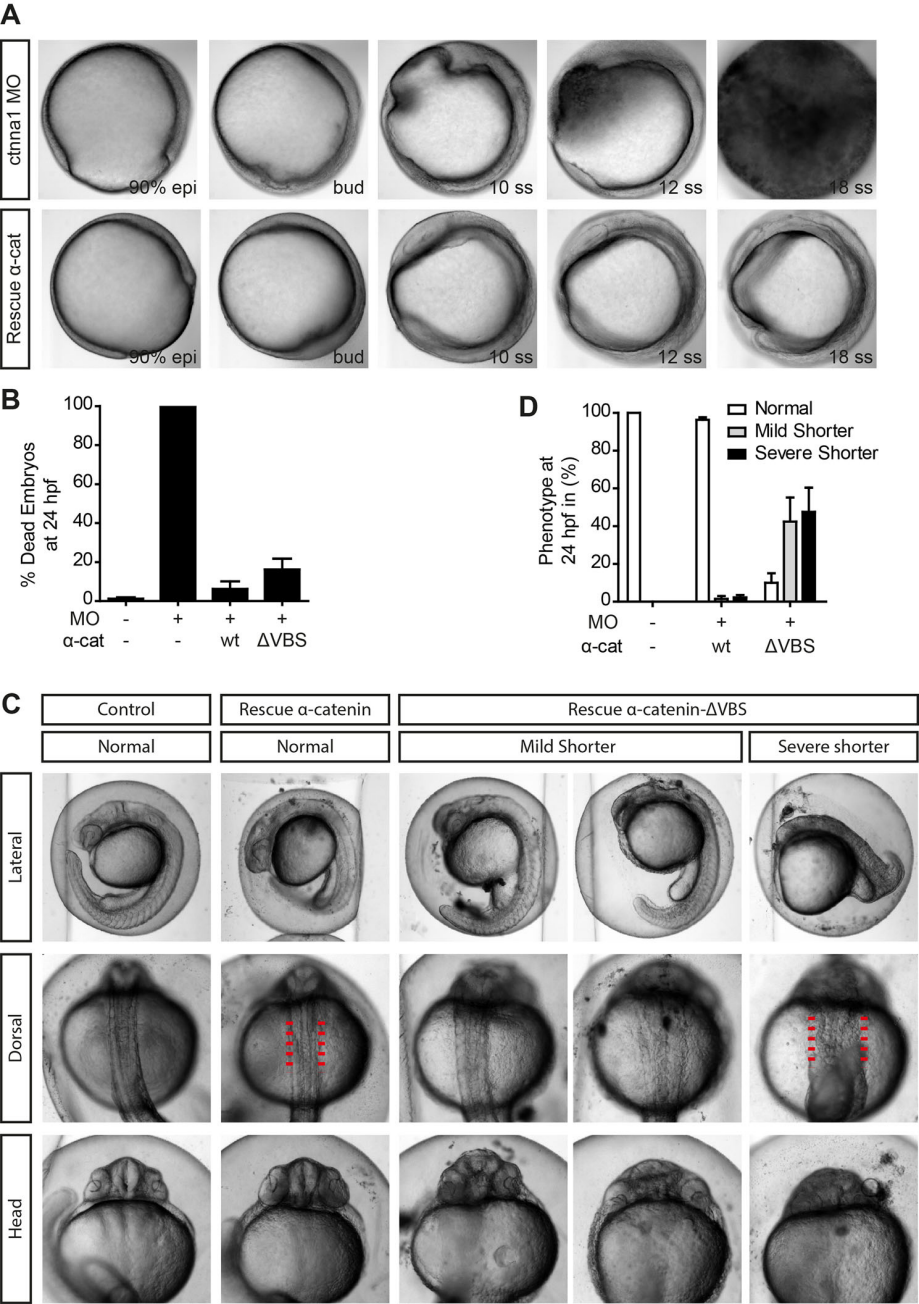
**The vinculin-binding domain is essential for proper body axis elongation.** (A) DIC images from a time-lapse image series of embryos injected with 0.84 ng *ctnna1* MO (top) rescued with 250 pg α-catenin-GFP (bottom). Embryos were imaged from 80% epiboly until 21 somite stage. (B,D) Quantification of the mortality (B) and the body axis phenotype (D) of non-injected embryos, embryos injected with 0.56 ng *ctnna1* MO or *ctnna1* MO-injected embryos rescued with either 250 pg α-catenin-GFP or 250 pg α-catenin-ΔVBS-GFP. Data represents three independent experiments, *n*>70 embryos per condition. Data is represented as the mean±s.e.m. (C) Representative images of non-injected control embryos (left) and *ctnna1* MO-injected embryos rescued with α-catenin-GFP or α-catenin-ΔVBS-GFP at 24 hpf. Clear morphological differences between α-catenin-GFP and α-catenin-ΔVBS-GFP-rescued embryos were apparent in anterior-posterior axis extension (top) as well as in the mediolateral dorsal convergence (middle and bottom). Red lines indicate the width of the dorsal body embryonic structure.

**Fig. 5.**
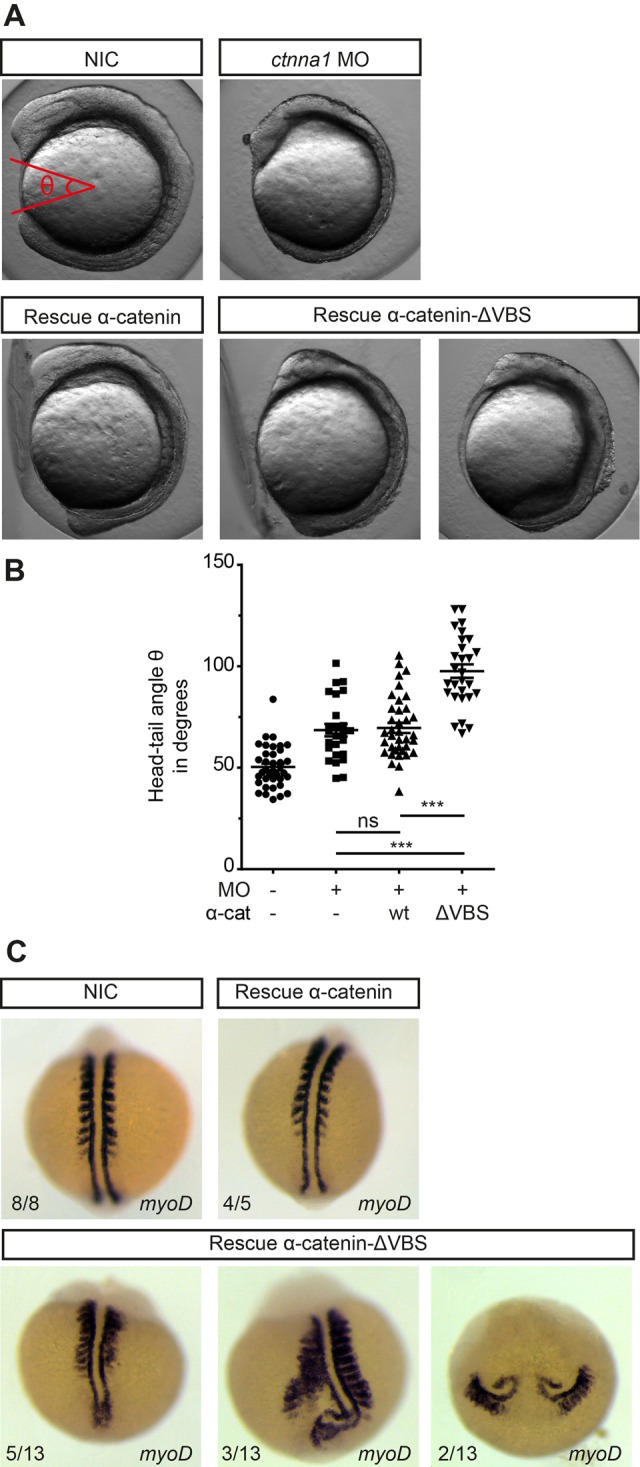
**Blocking vinculin recruitment to α-catenin induces convergent extension defects.** (A) DIC images of non-injected embryos, embryos injected with 0.84 ng *ctnna1* MO or *ctnna1* MO-injected embryos rescued with either 250 pg α-catenin-GFP or 250 pg α-catenin-ΔVBS-GFP at the 5-7 somite stage (12 hpf). (B) Quantification of the angle between the anterior-most point in the head, and the posterior-most point in the tail of the embryos (shown in red in A). Datapoints represent single embryos from three independent experiments (*n*>25 embryos per condition). Data is represented as mean±s.e.m. One-way ANOVA following Tukey's multiple comparison test was used for comparisons among multiple groups, with ***P<0.0001. (C) Dorsal views of whole-mount *in situ* hybridization at 9-11 somite stage of non-injected embryos and *ctnna1* MO-injected embryos rescued with either 250 pg α-catenin-GFP or 250 pg α-catenin-ΔVBS-GFP. *MyoD* marks the muscle precursors to visualize the somites.

Because the mutant *ctnna1* allele we generated is embryonic lethal in zygotic homozygous mutants, we could not establish an adult homozygous *ctnna1* mutant line. In addition, from the offspring of an incross of heterozygous *ctnna1* mutants, we could not distinguish the 25% homozygous mutants from their siblings before they started exhibiting the tissue rupture phenotypes described above. This negatively impacted our ability to analyze rescue experiments. To circumvent this problem, we opted to use anti-sense transcription-blocking *ctnna1* morpholino knockdown. By titrating the right amount of morpholino (Fig. S1A), we could recapitulate the embryo lysis phenotype observed in *ctnna1* zygotic mutants in almost 100% of the injected embryos. Similar to zygotic mutants, up until early somitogenesis, no severe developmental defects were obvious in the MO-injected embryos. A slight delay in development is apparent in all injected early-stage embryos, which is likely caused by the injection procedure (see Fig. 5A and Fig. S3 for images of MO-injected embryos). At a later stage during somitogenesis, *ctnna1* morphants injected with morpholino undergo embryo lysis just like zygotic mutants (Fig. 4A, top, still images from time lapse recordings). Embryo lysis could be prevented by transferring *ctnna1* morphants to isotonic Ringer's buffer at 50% epiboly and cells from the surviving morphants now exhibited a round morphology, similar to zygotic *ctnna1* mutants (Fig. S1B). Most importantly, we could rescue *ctnna1* morphants from embryo lysis by expressing either α-catenin-GFP (Fig. 4A,B) or α-catenin-ΔVBS (Fig. 4B) using mRNA injection at the one-cell stage. Together these results show that *ctnna1* morpholino-injected embryos exhibit a similar, αE-catenin-specific phenotype as *ctnna1* zygotic mutants.

Although *ctnna1* morphants rescued with α-catenin-ΔVBS were rescued from embryo lysis, they did show defects in anterior-posterior axis elongation after gastrulation. α-catenin-ΔVBS-GFP-rescued embryos often showed a shorter body axis than embryos rescued with α-catenin-GFP or wild-type, as assessed by eye at 1 dpf (Fig. 4C and Fig. S4A). For quantification and initial characterization, we grouped body axis elongation defects in two classes: mild and severe. These two classes could be clearly distinguished in α-catenin-ΔVBS rescued embryos but not in non-injected control and only sporadically in α-catenin-GFP rescued embryos. In the mild phenotype, in addition to the shorter body axis, some embryos also showed a flatter, wider head, as well as a broader dorsal body axis (Fig. 4C and Fig. S4A). Severely affected embryos show significant retardation in anterior-posterior body axis formation, in combination with a highly enlarged mediolateral body axis (Fig. 4C). These defects point to impaired convergent extension (CE) movements during gastrulation and have indeed been observed in embryos of well-established CE regulators such as Wnt/PCP signaling (Topczewski et al., 2001; van Eekelen et al., 2010). To rule out that these CE defects arose due to non-specific effects of co-injection of *ctnna1* morpholino with the mRNAs, we injected either α-catenin-GFP or α-catenin-ΔVBS-GFP mRNA in embryos from a heterozygous *ctnna1* mutant incross. Only injection of α-catenin-ΔVBS-GFP, but not wild-type α-catenin-GFP, induced defects in body axis elongation (Fig. S4B) further indicating that the observed defects arose due to the loss of the vinculin binding domain in α-catenin.

To further quantify the extent of CE defects, we measured the angle between the anterior and posterior ends of embryos at the 5-7 somite stage (12 hpf) (Fig. 5A). Although we did not observe strong defects in early body development in our visual inspection of *ctnna1* mutants (Fig. 2A), *ctnna1* morpholino-injected embryos did show an enlarged anterior-posterior angle compared to non-injected controls (Fig. 5B). We could not rescue this observed phenotype to control levels by expressing α-catenin-GFP mRNA and therefore conclude that it likely represents a slight delay in development caused by injection of the morpholino. Importantly, we observed a much more enlarged anterior-posterior angle in *ctnna1* morpholino-injected embryos expressing α-catenin-ΔVBS-GFP. These embryos also show reduced convergence of the somites to the midline compared to non-injected control embryos, or to *ctnna1* morphants expressing α-catenin-GFP (Fig. 5C and Fig. S4C). In addition, the notochords of more severely affected embryos show a wave-like morphology near the tailbud, while in the most affected embryos the complete structure of the notochord is misformed (Fig. 5C, bottom-middle and bottom-right respectively).

### Blocking cadherin mechanotransduction perturbs directionality of the lateral mesoderm during dorsal convergence

**Fig. 6.**
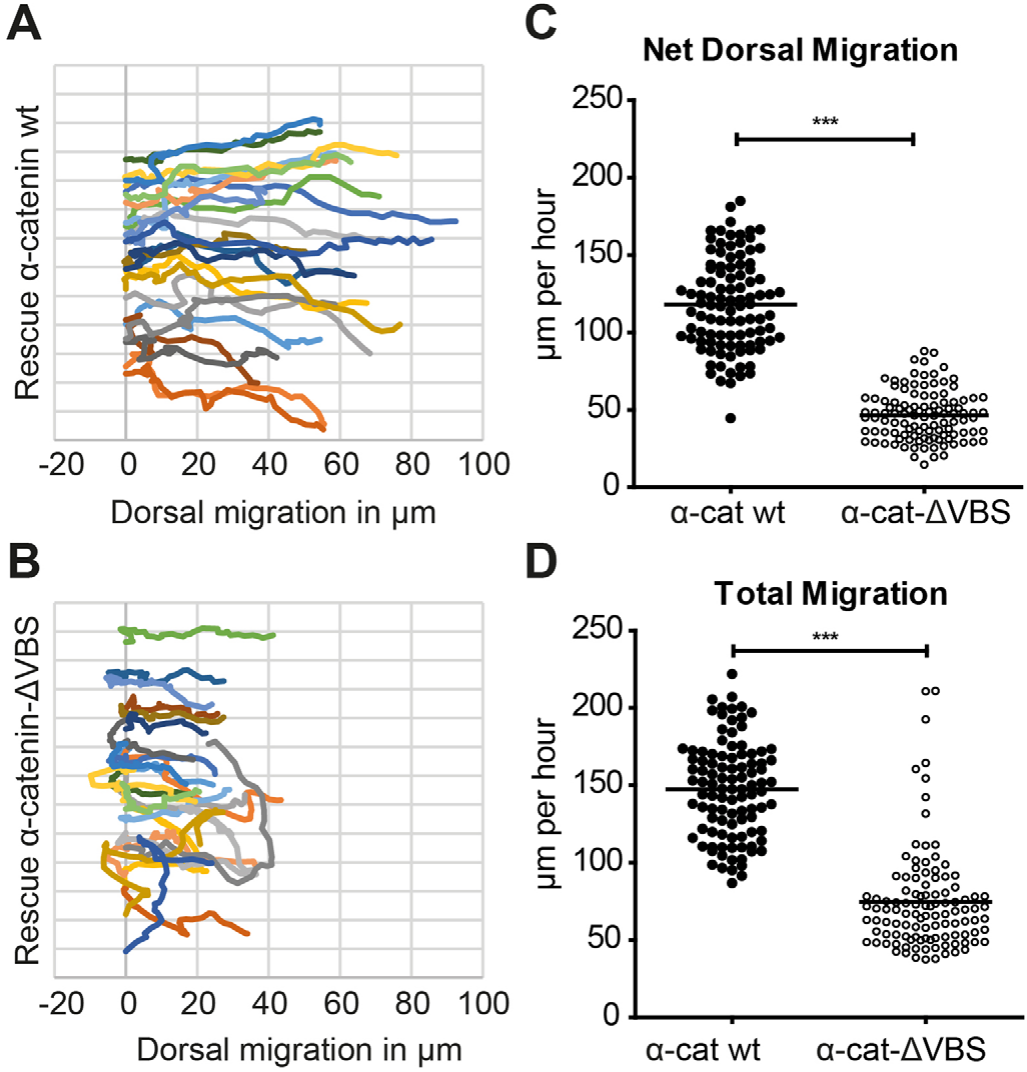
**Loss of cadherin mechanotransduction results in reduced dorsal convergence of the lateral mesoderm.** The dorsal convergence of the lateral mesoderm was imaged using DIC time-lapse microscopy. Lateral cells were imaged 90°C from the dorsal shield at 85-90% epiboly over 30 min with 45 s intervals. Embryos were injected with 0.84 ng *ctnna1* MO and rescued with either 250 pg α-catenin-GFP or 250 pg α-catenin-ΔVBS-GFP. (A,B) Representative migration tracks of individual lateral mesodermal cells (*n*=23) of a single embryo expressing α-catenin-GFP (A) or α-catenin-ΔVBS-GFP (B). Dorsal to the right and animal pole to the top. Tracks were offset along the dorsoventral axis such that each track starts at x=0. (C,D) Quantification of the net dorsal migration velocity (C) and the total migration speed (D) of lateral mesodermal cells. Data represent the mean of *n*>100 cells from 4 embryos per condition from 3 independent experiments (***=*P*<0.001, unpaired two-tailed *t*-test).

One major cadherin-dependent cell migration process underlying convergent extension is the dorsal convergence of the lateral mesoderm during late epiboly stages (Arboleda-Estudillo et al., 2010; Bakkers et al., 2004; von der Hardt et al., 2007). To test whether the widening of the dorsal body plan in *ctnna1* morphant embryos rescued with α-catenin-ΔVBS is correlated with impaired migration of the lateral mesoderm, we tracked individual mesodermal cells at approximately 90 degrees laterally from the dorsal midline during 85-90% epiboly stages. *Ctnna1* morphants expressing α-catenin-ΔVBS-GFP show a marked decrease in mesoderm cell migration compared to morphants expressing wild-type α-catenin-GFP, as shown in representative migration tracks (Fig. 6A,B and Movies 3 and 4). The quantification of the movement of these cells in Fig. 6C,D shows that both the net dorsal migration velocity as well as the total migration speed is significantly lower in α-catenin-ΔVBS-GFP-expressing morphant embryos in comparison to morphants expressing wild-type α-catenin-GFP. These results suggest that a reduction in dorsal migration of the lateral mesoderm cells contributes to the impairment of body axis formation in embryos in which cadherin mechanotransduction is perturbed.

**Fig. 7.**
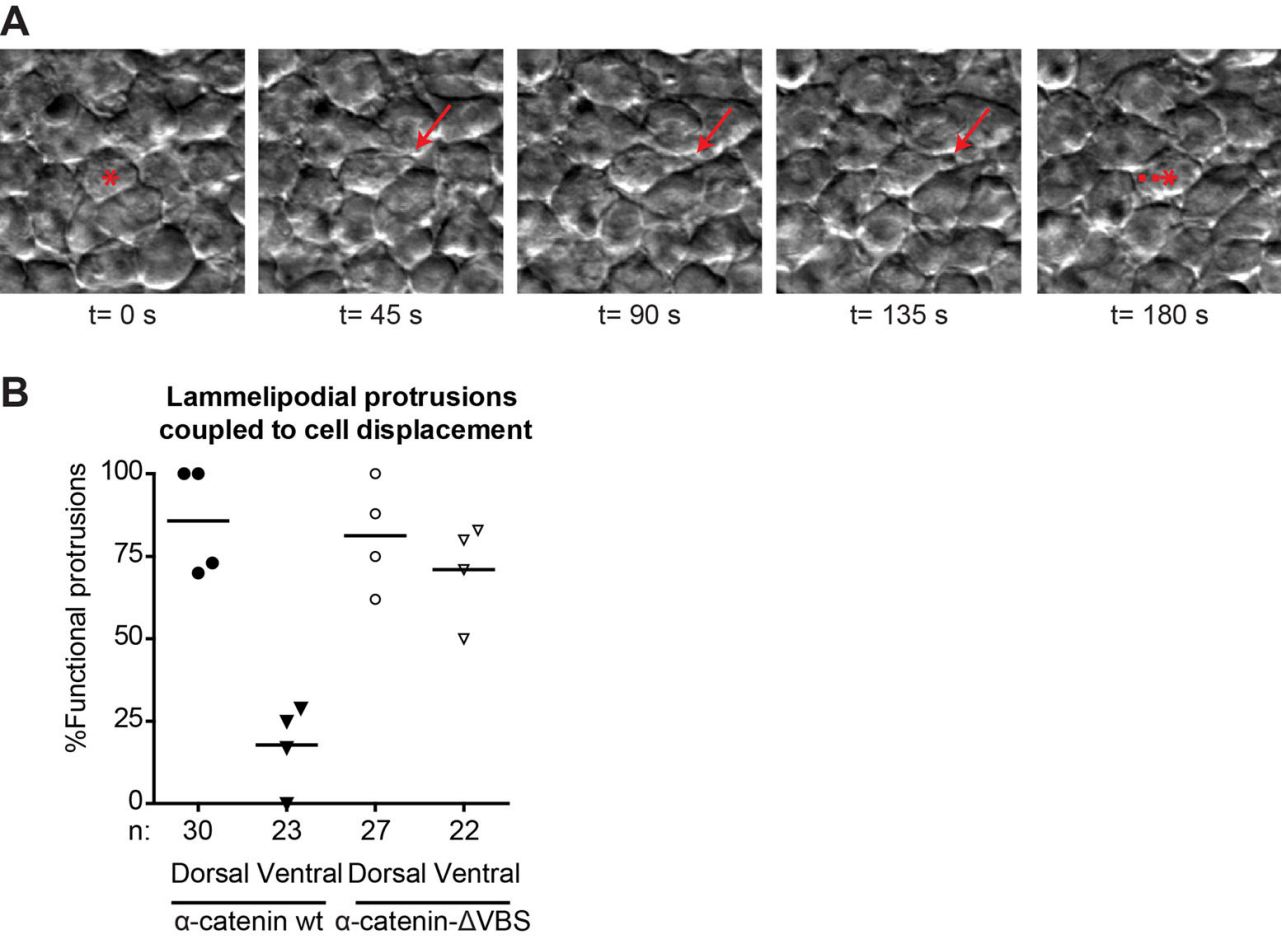
**Altered lammelipodial polarity of the lateral mesoderm after blocking cadherin mechanotransduction.** (A) Representative image sequence of a functional lamellipodial protrusion (red arrows) coupled to cell body displacement. The red asterisk marks the cell making protrusions, with the dashed lines indicating the displacement of the cell body. Dorsal to the right. (B) Percentage of lamellipodia coupled to cell body displacement in the direction of the protrusion by at least half the diameter of the cell. The number of cells analyzed (n) for each case is indicated below the x-axis. These were obtained from 4 embryos from 3 independent experiments and each datapoint in the graph represents the average value for one embryo analyzed.

The directional migration of the lateral mesoderm cells was found to be driven by lamellipodial protrusions. Whereas lamellipodia were found projected in all directions, productive lamellipodia (i.e. those that coincided with cell body movement) were predominant in the dorsal direction. A BMP-induced gradient in N-cadherin adhesion was hypothesized to cause the polarized productiveness of lamellipodia (von der Hardt et al., 2007). Supporting the hypothesis from this previous study, confocal imaging of lateral mesoderm cells labeled with α-catenin-GFP reveals the rapid formation and disassembly of cadherin junctions in mesoderm cells during dorsal migration (Movie 5). To directly investigate whether blocking cadherin mechanotransduction using α-catenin-ΔVBS also affects lamellipodia productiveness, we quantified the amount of lamellipodia that coincided with cell body displacements in either the dorsal or the ventral direction during lateral mesoderm migration at 85-90% epiboly. Similar to what has been reported before for wild-type embryos (von der Hardt et al., 2007), *ctnna1* morphants expressing wild-type α-catenin-GFP show approximately 85% functional lamellipodial protrusions in the dorsal direction (Movie 6), and only 20% in the ventral direction (Fig. 7A,B). In contrast, while morphants expressing α-catenin-ΔVBS show a similar amount of functional protrusions in the dorsal direction, the amount of functional protrusions in the ventral direction was strikingly increased to approximately 70%. Interestingly, the total frequency of protrusion formation was not observed to be different between morphants expressing α-catenin-GFP or α-catenin-ΔVBS-GFP, in either direction. These results indicate that loss of the mechanosensitive domain in α-catenin does not alter the formation of lamellipodia per se, but rather perturbs the dorsal polarization of functional lamellipodial protrusions.

## DISCUSSION

The cadherin complex present in adherens junctions is essential for the maintenance of epithelial tissue barriers. Cadherin complexes also regulate the rearrangements of cell-cell contacts between neighboring cells in multiple tissues to ultimately achieve proper tissue morphogenesis in the developing embryo. In this study we show that cadherin complex member αE-catenin is essential for epithelial barrier functioning and maintaining tissue shape during early morphogenesis. Furthermore, disruption of its vinculin-binding domain, which has been found to be important for force-induced junction reinforcement in cell culture, leads to altered lamellipodial polarization and defective dorsal migration of the lateral mesoderm during dorsal convergence movements. These results indicate that αE-catenin-dependent cadherin mechanotransduction is essential for the cell migration processes that underlie zebrafish morphogenesis.

We have used TALEN gene editing technology, targeting the *ctnna1* gene at exon 3, to generate a loss-of-function mutant called *ctnna1^hu10414^* (Fig. 1A). Gene disruption was verified by RFLP analysis and sequencing. We could not confirm the absence of α-catenin protein expression in *ctnna1^hu10414^* mutants (data not shown) for lack of suitable antibodies to detect zebrafish α-catenin on western blot. Nevertheless, based on its essential role as an adherens junction component, the resulting epithelial barrier phenotype is fully consistent with a lack of αE-catenin protein. In analogy, knockdown of α-catenin in epithelial MDCK cells (Twiss et al., 2012), as well as VE-Cadherin knockdown in endothelial HUVECs (Pannekoek et al., 2013) induced defects in barrier functioning as measured by trans-epithelial/-endothelial resistance. Many studies exist in which the crucial importance of cadherin-based cell-cell adhesion formation for epithelial barrier function was reported (Niessen, 2007). In addition, we were able to rescue the barrier defect by injecting α-catenin mRNA (Fig. 2E) and also anti-sense *ctnna1* morpholino knockdown closely phenocopies the mutant (Fig. 4A). Taken together this demonstrates that our mutant *ctnna1^hu10414^* is a true functional knockout, and is to our knowledge the first loss-of-function *ctnna1* mutant zebrafish line generated.

Tissue rupturing and subsequent embryo lysis in *ctnna1* mutant embryos occurred stochastically during somitogenesis (Fig. 2A-C). Based on previously described cadherin mutants and morphants, earlier severe defects in development would be expected if cells were unable to form cell-cell junctions from the start of development (Babb and Marrs, 2004; Kane et al., 2005; Shimizu et al., 2005). When we injected a translation-start-site-blocking *ctnna1* morpholinos we were able to phenocopy the mutant. At least two related α-catenin encoding genes exist in the zebrafish genome besides αE-catenin and it could well be that some functions of αE-catenin are rescued by redundancy with the products from these genes. In a previous study, however, the same morpholino was used with a tenfold higher concentration where it did induce epiboly delay (Schepis et al., 2012). Another explanation for a lack of strong defects in early development, as we derive our mutants by incrossing heterozygous parents, is that maternal contribution of mRNA and possibly protein provides sufficient α-catenin to sustain proper morphogenesis during early developmental stages. Tissue rupture is then observed in locations of the epithelium where the gradual decrease in functional α-catenin due to loss of zygotic expression has progressed the most. Similarly, at our used concentration, the morpholino may not deplete the maternally contributed α-catenin enough to induce epiboly defects, but may sufficiently inhibit translation of zygotic *ctnna1* transcripts to lead to the same embryo lysis phenotype also observed with the *ctnna1* mutant.

During the rescue experiments, it became clear that expression of both wild-type and α-catenin-ΔVBS by mRNA injection in *ctnna1* mutants/morphants rescued the barrier defect occurring during late somitogenesis stages. Whereas wild-type αE-catenin-rescued embryos now developed normally until at least 24 hpf, α-catenin-ΔVBS mRNA injection induced strong defects in convergence extension already in stages of development that appeared unaffected in mutants of MO-only injected embryos (Fig. 5). We also observed these convergence extension defects upon injection of α-catenin-ΔVBS in wild-type embryos in the absence of MO injection, although higher concentrations of mRNA were needed (data not shown). This apparent dominant effect of α-catenin-ΔVBS injection is consistent with the notion that proteins translated from the injected mRNAs gradually replace maternally supplied, or redundant, related α-catenin proteins in the cadherin complex in the developing embryos. Indeed, both wild-type α-catenin-GFP and α-catenin-ΔVBS-GFP localize to junctions already in early stage embryos (Fig. 3D, 8 hpf embryos). The fact that this does not lead to additional defects in the case of wild-type αE-catenin is expected. In the case of α-catenin-ΔVBS, however, this leads to a dominant perturbation of the VBS at cell-cell junctions, resulting in a loss of recruitment of vinculin and possibly other associated proteins. It is very likely that the loss of recruitment of VBS-associated proteins causes the observed defects in convergence extension. It should be noted that multiple proteins, including vinculin, α-actinin, afadin and formin have been found to associate with, or close to, the VBS of α-catenin and the perturbation of each of these or even additional unidentified binding partners could be the cause of the observed defects.

Convergent extension movements in zebrafish are driven by coordinated migration of mesoderm and overlaying ectoderm cells towards the dorsal midline, and by intercalations of deep cells into the enveloping layer (Tada and Heisenberg, 2012; Yin et al., 2008). Intercalation of deep cells is dependent on functional adherens junctions (Schepis et al., 2012) or E-cadherin (Babb and Marrs, 2004; Kane et al., 2005; Shimizu et al., 2005), and it is likely that blocking the α-catenin-vinculin interaction by α-catenin-ΔVBS negatively affected adherens remodeling and stabilization; however, whether this contributed to the observed phenotypes remains to be investigated. We did find that dorsal convergence of the lateral mesoderm was strongly reduced when we perturbed cadherin mechanotransduction using α-catenin-ΔVBS (Figs 5C and 6). A previous study showed that this coordinated cell migration process is dependent on E-cadherin, as this migration is reduced in both E-cadherin MO knockdown and E-cadherin/*weg* mutant embryos (Arboleda-Estudillo et al., 2010). Another study showed that this migration is also dependent on N-cadherin, which is negatively regulated by a ventral-to-dorsal Bmp gradient to generate a reverse gradient in cadherin adhesion from dorsal to ventral regions (von der Hardt et al., 2007). Dorsal convergence of the paraxial mesoderm was also affected in N-Cadherin/*bib* mutants (Warga and Kane, 2007). Our results now show that the presence of cadherin adhesions is not only needed, but that also their mechanotransduction capacity is essential for the proper migration of the lateral mesoderm cells towards the dorsal midline.

Besides the above mentioned studies implicating cadherin adhesion in dorsal convergence in zebrafish embryos, perturbation of myosin IIB was found to disturb convergence in *Xenopus* embryos (Skoglund et al., 2008) and it was argued that the failure to generate polarized cytoskeletal forces was the main cause of the observed defects. It is conceivable that polarized organization of the cytoskeleton depends also on its anchoring to the cellular cortex, which is mediated for a large part by adhesion complexes. In *Xenopus*, both integrin adhesions as well as cadherin adhesions have been implicated in convergence cell movements (Weber et al., 2011); in zebrafish there is little evidence for involvement of integrin adhesion. In general it is clear that both integrin as well as cadherin adhesions generate feedback signals that organize cellular cytoskeletons in response to mechanical interactions with the extracellular environment (Leckband and de Rooij, 2014). As we have perturbed α-catenin, which is a cadherin-specific cytoskeletal linker, it is likely that we have directly impacted on the feedback signals from cadherin adhesion to cytoskeletal organization, which may have impacted integrin-dependent adhesion and migration processes indirectly (Barry et al., 2015).

Repeating the detailed assessment of mesoderm cell migration by Von der Hardt et al. (2007), we find an increasing number of functional lamellipodial protrusions in the ventral direction, resulting in loss of migration polarity in α-catenin-ΔVBS-expressing embryos (Fig. 7B). This indicates that the mechanosensitive domain is important for the polarization of lamellipodial functionality in the dorsal direction. As discussed above, a likely explanation is that a polarized, force-dependent recruitment of vinculin and/or another VBS-interacting protein normally initiates a positive feedback mechanism that reinforces adhesions, reorganizes the associated cytoskeleton and generates traction in dorsal lamellipodia preferentially. A gradient of tissue stiffness along the ventral-dorsal axis could establish such polarization. Whereas the Hammerschmidt work (Von der Hardt et al., 2007) suggests interactions among deep cells within the same layer orchestrate polarized movements, it is to be expected that cadherin-mediated interactions between mesoderm cells and other cell layers (epiblast or EVL, or yolk syncytial layer) also contribute (Carvalho and Heisenberg, 2010). Mechanosensitive feedback, establishing a correlation between pro-migratory traction generation and substrate stiffness, has been observed for N-cadherin-based adhesions in a tissue culture model (Ladoux et al., 2010). Related, tension-regulated morphogenetic processes have been hypothesized for *Drosophila* border cell migration, in which the front/back polarity of border cell clusters is maintained through higher tension on E-Cadherin at the front of the cluster, which in turn polarizes Rac activity in the same direction (Cai et al., 2014), and in the migration of cells in the prechordal plate, where directionality of the cell collective may depend on mechanical forces transmitted at E-cadherin contacts (Dumortier et al., 2012). Future studies will have to determine which cues could contribute to the initial polarization of the lateral mesoderm. The fact that both BMP signaling as well as cadherin mechanotransduction contribute to mesoderm convergence underscores the notion that tissue morphogenesis is the combined result of regulation by chemical and mechanical cues.

In conclusion, the morphogenetic defects induced here by α-catenin-ΔVBS in zebrafish gastrulation provide direct evidence for the hypothesis that α-catenin-dependent cadherin mechanotransduction is essential for the proper development of a living embryo. Our experiments furthermore show how mesoderm cell migration is one process controlled by cadherin mechanotransduction. Future studies using the tools developed may elucidate additional aspects of zebrafish morphogenesis controlled by forces at cadherin junctions.

## MATERIALS AND METHODS

### Fish lines and husbandry

The following fish lines were used: Tupfel Longfin (TL) and *ctnna1^hu10414^*. Fish were maintained according to standard laboratory conditions. Animal experiments were approved by the Animal Experimentation Committee (DEC) of the Royal Netherlands Academy of Arts and Sciences.

### Cell lines and cell culture

DLD1 R2/7 α-catenin-deficient colon carcinoma cells (a gift from Frans van Roy, Ghent University, Ghent, Belgium) were cultured in high glucose DMEM containing L-glutamine, supplemented with 10% fetal calf serum and penicillin/streptomycin in standard 10 cm culture dishes. Cells were transfected with zebrafish α-catenin-GFP or α-catenin-ΔVBS-GFP using X-tremegene9 (Roche) as per manufacturer's instructions. Cells were tested negative for mycoplasma infection.

### Construction of zebrafish α-catenin-GFP constructs

Zebrafish α-catenin was kindly provided to us by James Nelson (Stanford University). We generated a GFP-tagged version by generating a PCR fragment containing XhoI/XbaI restriction sites at the terminal ends using the following primers: forward 5′- GACACTCGAGCCATGACGAGCATTAACACTGCTAACATC-3′ and reverse: 5′- GACATCTAGACGGAGGAGAGTAGATTAGACTTA-3′. We then digested the PCR product with XhoI and XbaI, and the pEGFP parental vector with SalI and XbaI (Clontech), after which we ligated the product, resulting in ZFα-catenin-GFP x pEGFP. We then subcloned α-catenin-GFP into pCS2 vector for mRNA transcription. To achieve this we generated a PCR product using the following primers: forward 5′- CGATTCGAATTCAAGGCCTCGCCACCATGGTGAGCAAG-3′ and reverse 5′- CGTAATACGACTCACTATAGTTTCAAATACTATCCATAGCTTTGAACTCGCTCAG-3′. The PCR product was then ligated into pCS2 digested with XhoI and XbaI using Gibson Assembly (NEB), resulting in zebrafish α-catenin-GFP in pCS2.

We generated zebrafish α-catenin-ΔVBS-GFP by swapping the ΔVBS domains (which originate from chicken vinculin) with our previously described murine α-catenin-ΔVBS (Huveneers et al., 2012). We first generated a PCR fragment from our B141 α-catenin-ΔVBS-GFP vector using the following primers: forward 5′- CGAGGATTCCAGCTTCTCCCAGACTGCAGGTGGAGGAGAGCTGGCATACGCT-3′ and reverse 5′-GTTAGGTTTGGCAGCCAGGGCCAAAGCTGCATTAATCACCTGAGGACACAGCGCTTCTAACTG-3′. We then digested the zebrafish α-catenin-GFP x pCS2 vector using Bsu36I and NheI restriction sites, after which we ligated the PCR product using Gibson Assembly, resulting in zebrafish α-catenin-ΔVBS-GFP in pCS2.

Plasmids were linearized with NotI (Promega) after which IVT mRNA was generated using the mMessage mMachine SP6 kit (Ambion), and purified using the RNeasy mini kit (Qiagen).

### Immunohistochemistry and cytoskeletal washout

For immunocytochemistry, DLD1 R2/7 cells were washed 1 time in PBS, 1 time in CSK buffer (300 mM Sucrose, 0.5% TX-100, 10 mM Pipes pH 7, 50 mM NaCl, 3 mM CaCl2, 2 mM MgCl2) and 1 time in PBS before fixation using 4% paraformaldehyde in PBS for 20 min. After fixation, cells were permeabilized with 0.4% Triton X-100 in PBS for 5 min and blocked in 2% BSA for 1 h. Phalloidin-415 (Promokine), vinculin mouse primary antibody (hVin1 clone 1:500; Sigma) and Alexa-Fluor-594 secondary antibody (Life Technologies) were diluted in 2% BSA and incubated with the cells for 1 h. Afterwards, cells were mounted in Mowiol 4–88/DABCO solution (Sigma-Aldrich).

Embryos were fixed in 2% PFA in PBS overnight at 80% epiboly stage. After washing in PBS, embryos were dechorionated and further washed 3-5 times for 5 min in PBT (PBS+0.1% Triton-X100). Embryos were blocked in PBT+10% Normal Goat Serum for 1 h, then incubated O/N with Phalloidin-Alexa-Fluor-594 (Life Technologies). Samples were then washed 4×30 min in PBT before mounting in 0.3% agarose in E3 embryo medium.

### Generation of *ctnna1* TALEN constructs

TALENs targeting the *ctnna1* locus were designed using the TALE-NT tool (https://tale-nt.cac.cornell.edu/node/add/talen) using the guidelines specified in Cermak et al. (2011). The chosen optimal target sequence in exon 3: 5′- TTGTGTTTATTGCAGGTCACCACACTTGTGAACTCCAGCAACAAAGGTCCA-3′ (binding sites of the left and right TALEN arms are underlined) contains a HphI (NEB) restriction enzyme site (GGTGA). TALENs were generated using the Golden Gate kit (Cermak et al., 2011) in combination with the obligate heterodimeric FokI pCS2TAL3DD (Addgene, #37275) and pCS2TAL3RR (Addgene, #37276) backbones (Ota et al., 2013).

TALEN targeting plasmids were linearized with NotI (Promega) after which IVT mRNA was generated using the mMessage mMachine SP6 kit (Ambion), and purified using the RNeasy mini kit (Qiagen).

### Microinjection of morpholinos and mRNA

Translation-blocking morpholinos (MOs) targeting zebrafish *ctnna1* were synthesized by GeneTools, LLC. The MO partially blocked the 5′UTR and ATG-start codon: 5′-TAATGCTCGTCATGTTCCAAATTGC-3′ and is described earlier (Schepis et al., 2012). Both MOs and mRNA were diluted in nuclease-free water containing phenol-red. Injection mix was microinjected into the yolk at the one-cell stage. Rescue experiments were performed by sequential injection of *ctnna1* MO and α-catenin mRNA.

### Genotyping of mutant alleles

To isolate genomic DNA, zebrafish embryos or fin clips were incubated in 50 µl or 100 µl embryo lysis buffer respectively, for 60 min at 60°C. Proteinase K was heat-inactivated by incubating the samples for 15 min at 95°C. The genomic DNA was used as template to amplify the *ctnna1* TALEN target site. Primers flanking the target site of *ctnna1*: Alphacat HphI forward, 5′- GTCTTGCCACCCCTTAATCC-3′ and Alphacat HphI reverse, 5′- CAATCTTCTCGCCTTTCTCC-3′.

To assess *ctnna1* mutant alleles, the PCR amplicons were incubated with HphI (NEB) at 37°C for 2 h, resulting in the generation of 245 bp and 140 bp fragments. Successful mutagenesis was detected by loss of the restriction recognition site and the presence of an undigested band of 385 bp. Mutant alleles were sequenced using primer Alphacat HphI reverse, 5′- CAATCTTCTCGCCTTTCTCC-3′.

### *In situ* hybridization

Embryos were fixed overnight in 4% paraformaldehyde (PFA) and stored in MeOH. *In situ* hybridization experiments were performed as previously described (Nöel et al., 2013). Once staining had developed, embryos were washed several times in phosphobuffered saline with 0.1% tween and fixed overnight in 4% PFA.

### Microscopy and live imaging

Fixed cells were imaged using an inverted research widefield microscope (Eclipse Ti; Nikon) with perfect focus system, equipped with a 60×1.49 NA Apochromat total internal reflection fluorescence (oil) objective lens, a microscope cage incubator (OkoLab), and an EM charge-coupled device (CCD) camera (Andor Technology) controlled with NIS-Elements Ar 4.0 software (Nikon). All widefield images, unless specifically indicated otherwise were background subtracted by ImageJ's rolling ball (r=20) and sharpened for display by Fiji/ImageJ's unsharp mask filter (r=1, weight=0.6).

Fixed embryos were imaged on the same microscope using the Nikon C1 Confocal system. For time-lapse imaging, embryos were dechorionated at 50% epiboly stage and embedded in 0.3% agarose in E3 embryo medium. DIC images were taken every 15 min on the same microscope using a 10×0.3 NA Plan Fluor objective lens (Nikon). Embryo staging was determined by wild-type embryos imaged simultaneously with mutants or morphants. Live embryos at 12-24 hpf and fixed embryos after whole-mount *in situ* hybridization were imaged using a Leica MZFLIII upright microscope.

### Quantification of body axis elongation

Embryos were imaged from the lateral position at 12 hpf. Images were processed in Fiji (ImageJ) as follows: the center of the embryo was determined by manually fitting a circle over the yolk. Lines were drawn from the center to the most anterior position of the head, and most-posterior position of the tail. The head-tail angle was then assessed using the angle tool.

### Imaging dorsal convergence and lamellipodia

Embryos were dechorionated at 50% epiboly stage and embedded in 0.3% agarose in E3 embryo medium. DIC images of the lateral mesoderm during dorsal convergence (85-90% epiboly) were taken every 45 s on a Nikon Ti microscope using a 20×0.75 NA Plan Fluor objective lens. Cells residing in the layer closest to the yolk syncytium were tracked using Fiji/imageJ with the Manual Tracking plugin. Lateral mesodermal cells from wild-type embryos overexpressing α-catenin-GFP were imaged during dorsal convergence (85-90% epiboly) using a Leica SP8 confocal microscope. Images were taken every 45 s. Images were sharpened for display by using Fiji/ImageJ's Gaussian blur filter (r=2) and unsharp mask filter (r=2, weight=0.6).
